# The *Plasmodium* PI(4)K inhibitor KDU691 selectively inhibits dihydroartemisinin-pretreated *Plasmodium falciparum* ring-stage parasites

**DOI:** 10.1038/s41598-017-02440-6

**Published:** 2017-05-24

**Authors:** L. Dembele, X. Ang, M. Chavchich, G. M. C. Bonamy, J. J. Selva, M. Yi-Xiu Lim, C. Bodenreider, B. K. S. Yeung, F. Nosten, B. M. Russell, M. D. Edstein, J. Straimer, D. A. Fidock, T. T. Diagana, P. Bifani

**Affiliations:** 1 Novartis Institute for Tropical Diseases, 10 Biopolis Road, #05-01 Chromos, 138670 Singapore, Singapore; 2 Department of Drug Evaluation, Australian Army Malaria Institute, Brisbane, QLD 4051 Australia; 3 Shoklo Malaria Research Unit, Mahidol-Oxford Tropical Medicine Research Unit, Faculty of Tropical Medicine, Mahidol University, Mae Sot, Thailand; 4 Centre for Tropical Medicine and Global Health, Nuffield Department of Medicine, University of Oxford, Oxford, UK; 5 Department of Microbiology and Immunology, University of Otago, Dunedin, New Zealand; 6 Department of Microbiology and Immunology, Columbia University Medical Center, New York, NY 10032 USA; 7 Division of Infectious Diseases, Department of Medicine, Columbia University Medical Center, New York, NY 10032 USA; 8 Department of Microbiology and Immunology Program, Yong Loo Lin School of Medicine, Life Sciences Institute, National University of Singapore, 119077 Singapore, Singapore

## Abstract

Malaria control and elimination are threatened by the emergence and spread of resistance to artemisinin-based combination therapies (ACTs). Experimental evidence suggests that when an artemisinin (ART)-sensitive (K13 wild-type*) Plasmodium falciparum* strain is exposed to ART derivatives such as dihydroartemisinin (DHA), a small population of the early ring-stage parasites can survive drug treatment by entering cell cycle arrest or dormancy. After drug removal, these parasites can resume growth. Dormancy has been hypothesized to be an adaptive physiological mechanism that has been linked to recrudescence of parasites after monotherapy with ART and, possibly contributes to ART resistance. Here, we evaluate the *in vitro* drug sensitivity profile of normally-developing *P. falciparum* ring stages and DHA-pretreated dormant rings (DP-rings) using a panel of antimalarial drugs, including the *Plasmodium* phosphatidylinositol-4-OH kinase (PI4K)-specific inhibitor KDU691. We report that while KDU691 shows no activity against rings, it is highly inhibitory against DP-rings; a drug effect opposite to that of ART. Moreover, we provide evidence that KDU691 also kills DP-rings of *P. falciparum* ART-resistant strains expressing mutant K13.

## Introduction

Since the introduction of ACTs in 2000, there has been mounting evidence of *P. falciparum* resistance to ART derivatives in Southeast Asia, manifesting through longer parasite clearance times in patients^1, 2^. In 2008, the first indications of a rise in ACT treatment failure rates were reported for dihydroartemisinin-piperaquine (DHA-PPQ) and artesunate-mefloquine (AS-MQ) combinations in western Cambodia^3–5^. Since then, resistance to ACTs has spread and is now established in Myanmar, Thailand, and Vietnam^6, 7^. Longer parasite clearance times have been linked to decreased susceptibility to ART at the very early post-erythrocyte invasion ring stages^8–10^. Compelling evidence has also linked clinical ART resistance to point mutations in the propeller domain of the *P. falciparum* K13 (Kech13) protein^10, 11^. Mutations in the *K13* gene confer increased ring-stage survival *in vitro* ^12^.

The very short elimination half-life of ART in patients has been a frequent explanation for the high recrudescence rates observed with this class of drugs when used as monotherapy^13, 14^. However, drug-induced dormancy or quiescence^15, 16^ have also been proposed as potential contributor to parasite recrudescence and host treatment failures^13, 17–19^. Studies suggest that upon exposure to ART or more specifically its active metabolite DHA, a sub-population of ART-sensitive *P. falciparum* ring stage parasites can undergo a temporary growth arrest (i.e. dormancy)^20, 21^ that allows them to survive drug exposure until this pressure is removed and normal growth can resume^18, 22^. It remains to be determined whether this phenomenon is a drug-induced stress response or instead is due to the presence of a pre-existing sub-population of phenotypically drug-resistant “stalled” parasites^23^. Notably, a stress response may be enhanced in parasites bearing the K13-propeller mutations, which could result in an increase of this sub-population in ART-resistant parasites^11^.

Although detailed descriptions of morphological and mitochondrial activity of dormant parasites have been reported^24, 25^, no specific molecular or phenotypic markers have to date been identified that can differentiate rings from DHA-pretreated rings (DP-rings). These DP-rings, reported as pyknotic-like, have been described as either non-viable parasites^11^ or viable rings capable of resuming growth^24^. Others have described DP-rings with a compact cytoplasm^16^ that can be stained with the mitochondrial vital dye Rhodamine 123^24^. These parasites have been considered viable and capable of re-entering the life cycle^24^.

Previously, we reported the *Plasmodium* PI4K-specific inhibitor KDU691, an imidazopyrazine with potent anti-parasitic activity against blood stage schizonts, gametocytes and liver stages^26^. However, KDU691 did not show activity against rings^26^. PI4K is predicted to be responsible for membrane trafficking in key stages of the *Plasmodium* life cycle including the asexual blood stages, specifically during the period of schizont maturation prior to merozoite egress from the infected red blood cell^26^.

In the present study, we evaluated the activity of KDU691 against DP-rings. For comparison, we also evaluated a panel of standard antimalarials. This included tafenoquine (TQ) that is active against both blood and liver stage parasites including hypnozoites^27^, atovaquone (ATQ) that inhibits red blood cell and hepatic schizonts, and lumefantrine (LUM) that is active only against asexual blood stages^28^. We report that KDU691 selectively kills DP-rings of the *P. falciparum* W2-WT strain, Dd2-WT, derivative transgenic parasite lines bearing K13 mutations that are casual for ART resistance *in vitro* ^12, 14^ and *P. falciparum* clinical isolates.

## Results

### Rings pre-treated with DHA survived and are sensitized to PI4K inhibitors

**Figure 1:**
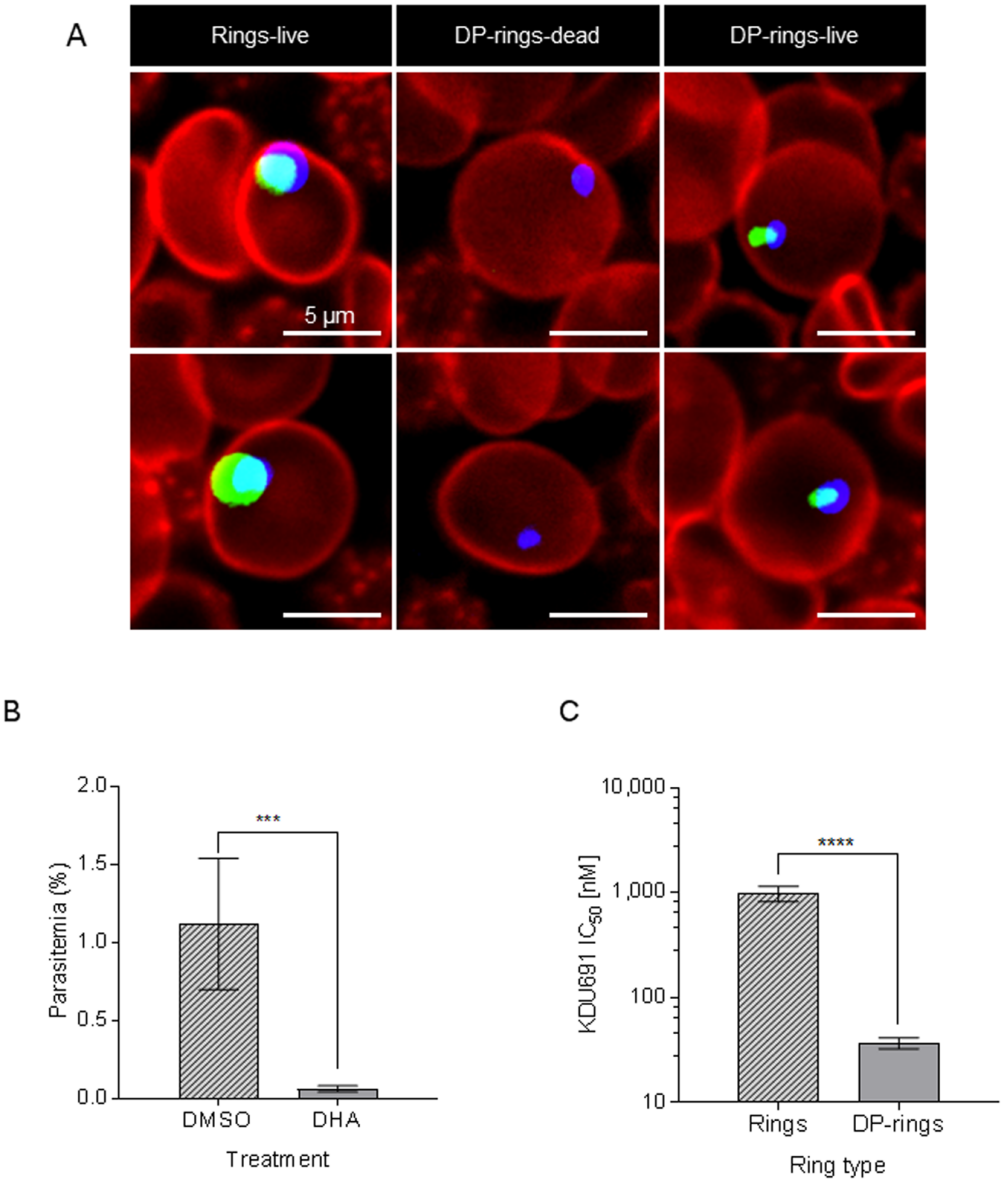
Rings pre-treated with DHA are sensitized to the PI4K inhibitor KDU691. (**A**) Determination of live (green-blue) and dead (blue) parasites by high content imaging (HCI) from rings (3–6 hours) and DP-rings (18 hours following 6 hours, 700 nM DHA treatment). Blue (DAPI): DNA; Green (MitoTracker® Orange): functional mitochondria; Red: (wheat germ agglutinin (WGA) conjugated to Alexa Fluor® 647): red blood cells. (**B**) Mean parasitemias are shown for ring-stage parasites at 24 hours after exposure to 700 nM DHA for six hours. (**C**) IC_50_ of KDU691 against rings and DP-rings following treatment for 22 hours. In A and B live parasites were stained with Rhodamine 123 and quantified with flow cytometry. Data for B and C (means ± SEM) were calculated from three independent biological experiments.

DHA pre-treated rings (DP-rings) displayed distinctive morphology with a “pyknotic-like” appearance (Fig. 1A and Supplementary Fig. S1). Unlike dead rings, viable DP-rings were also characteristically stained with the mitochondrial markers MitoTracker® Orange, a dye used to evaluate mitochondrial membrane potential in viable cells (Fig. 1A). In addition, DP-rings showed delayed growth while rings continued unrestricted development into trophozoites, schizonts and second (or next)-generation rings (Supplementary Fig. S1). When synchronized cultures of *P. falciparum* W2 rings at a starting parasitemia of 1% were treated for six hours with 700 nM DHA, only about 0.07% parasitemia, corresponding to 7% apparently surviving parasite, were detected after 24 hours by Rhodamine 123 staining and flow cytometry analysis (Fig. 1B). DP-rings proved to be 20–fold more susceptible than DHA-untreated rings to the PI4K inhibitor KDU691 (Fig. 1C).

### Selective inhibition of DP-rings appears to be specific to PI4K inhibitors

**Figure 2:**
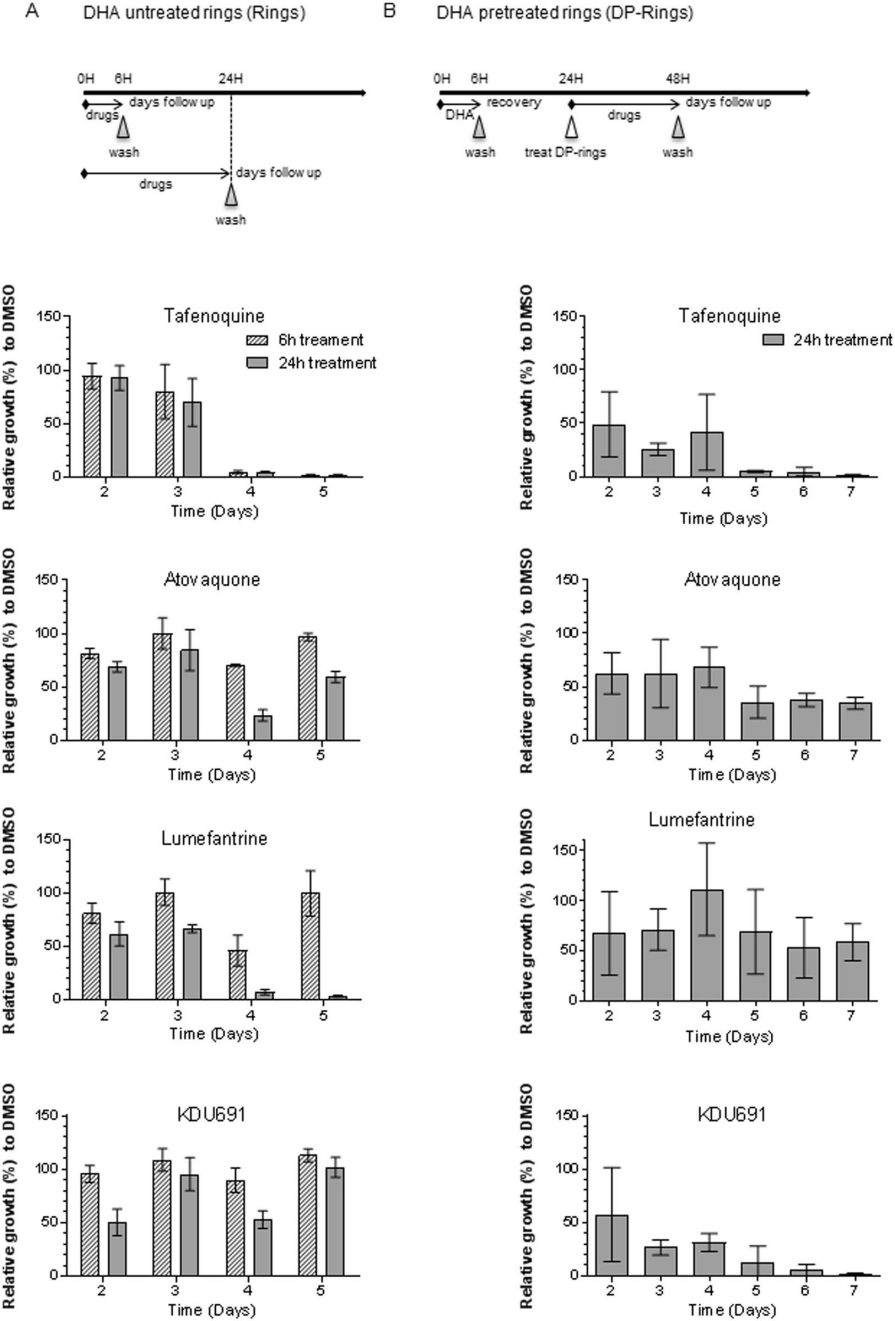
Selective inhibition of DP-rings appears to be specific to PI4K inhibitors. (**A**) Synchronized rings treated at concentrations corresponding to the IC_90_ of TQ (0.7 µM), ATQ (0.003 µM), LUM (0.06 µM), KDU691 (0.7 µM) and untreated control for six or 24 hours. Growth was monitored by HCI. (**B**) Dormancy was induced by exposing synchronized rings to 700 nM DHA for 6 hours. After washing and a further 18 hours of culture, the DP-rings were treated with the same panel of drugs and concentrations as for panel A for 24 hours. Parasite growth was monitored for seven days by HCI. For the DP-rings, growth in the presence of drugs (applied during the period of 24 to 48 hours) was normalized to the growth of parasites not exposed to drugs during the same period. Percent growth was measured by HCI using MitoTracker® Orange relative to DMSO. Data are from three biological experiments with technical duplicates (mean ± SEM % growth).

Following initial observations that DP-rings proved to be more susceptible to KDU691 (Fig. 1C), a thorough comparative analysis of the inhibitory activities of TQ, ATQ, LUM and KDU691 were evaluated against normal rings and DP-rings. The selected panel of drugs is active against both asexual blood and liver stages of the *Plasmodium* life cycle and includes TQ, which is also active against *P. vivax* hypnozoites^27^. Parasites (DP-rings and rings) were subjected to drug pulses at concentrations corresponding to their respective 72 hours SYBR Green assay IC_90_ values (Fig. 2). Unlike the ring-stage survival assay (RSA_0-3h_)^8–10^, which utilizes zero to three hours old rings, our experiments were performed using three to six hours old rings. Briefly, sorbitol-synchronized DHA-untreated three to six hours old rings were exposed to test drugs for six or 24 hours (Fig. 2A). DHA-pretreated DP-rings were exposed to test drugs for 24 hours, i.e. 18 hours after the six hours of 700 nM DHA pre-treatment (Fig. 2B). Viability was measured using MitoTracker® Orange and the drug susceptibility profile of the DP-rings was determined as previously described^13, 14^ (Supplementary Fig. S2A,B). TQ (700 nM) proved to be highly active on both rings and DP-rings (Fig. 2A, B). This completely inhibited rings after six or 24 hours of exposure as well as DP-rings after 24 hours of exposure. ATQ (3 nM) did not inhibit rings with either six or 24 hours of exposure and inhibited ~50% of DP-rings. Treatment of the rings with LUM (60 nM) proved to be inhibitory only after 24 hours of exposure and showed poor activity on DP-rings. In agreement with previous studies^26^, KDU691 (700 nM) had no inhibitory activity on rings, even after 24 hours of exposure, and parasites fully recovered by day five. In contrast, DP-rings treated for 24 hours with KDU691 were potently inhibited and did not recover, even after day five (Fig. 2). Taken together, these data provided evidence that KDU691 selectively kills DP-rings and suggest that this property is not commonly shared with other antimalarial drugs.

### KDU691 mechanism of action on DP-rings is dependent on the PI4K signaling pathway

**Figure 3:**
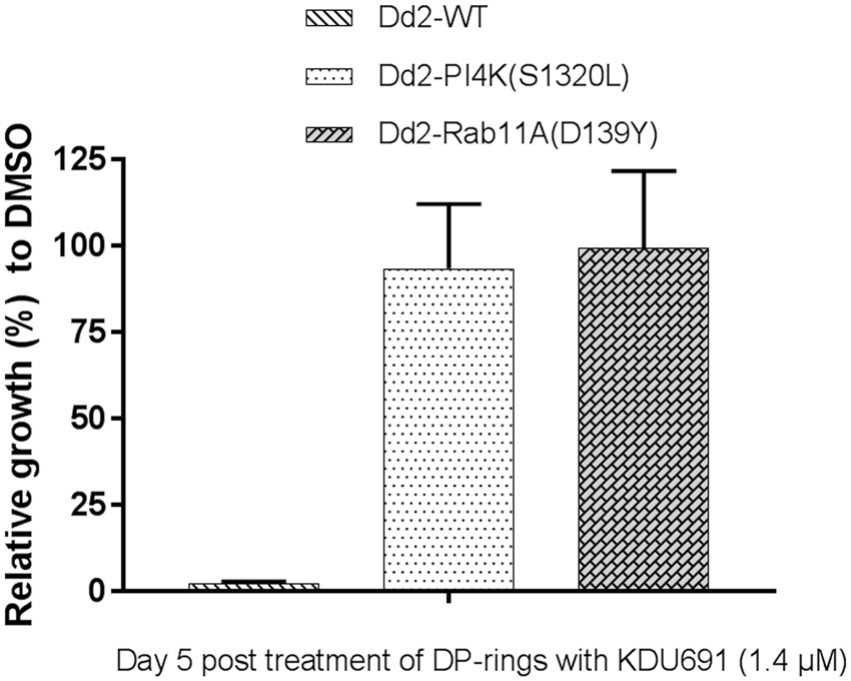
KDU691 mechanism of action on DP-rings is dependent on the PI4K signaling pathway. Mean+ SEMS results were from three biological experiments with three technical replicates each. Percent growth was measured by HCI using MitoTracker® Orange relative to DMSO.

To confirm that KDU691 inhibits DP-rings by targeting the *Plasmodium* PI4K pathway, we evaluated its activity against DP-rings of the two previously reported KDU691-resistant transgenic strains that were engineered on a Dd2 background, namely Dd2*-PfPI4K-S1320L* and Dd2*-PfRab11A-D139Y* ^26^. In a standard 72-hours drug assay the wild-type (WT) Dd2 strain showed a KDU691 IC_90_ of 1.4 µM, while these mutations respectively confer a five and four fold shift^26^. At this concentration of 1.4 μM, KDU691 efficiently inhibited Dd2-WT DP-rings but had no activity against the Dd2-WT rings (Fig. 3; Supplementary Fig. S3A). In contrast, 1.4 μM KDU691 had no inhibitory activity on the recovery of Dd2*-PfPI4K-S1320L* and Dd2*-PfRab11A-D139Y* DP-rings and rings, which both fully regained growth by day 5 (Fig. 3 and Supplementary Fig. S3A). Importantly, we showed that TQ (IC_90_: 1.4 μM) inhibited both rings and DP-rings of the PI4K-resistant Dd2 transgenic strains on days three (Supplementary Fig. S3B) and four (Supplementary Fig. S3C). These data provide evidence that the KDU691 mechanism of action operating selectively in the DP-rings is dependent on the *Plasmodium* PI4K signaling pathway.

### K13-mediated ART resistance seems to correlate with a higher fraction of DP-rings that remain sensitive to PI4K inhibition

Having established that DP-rings can be chemically discriminated from rings with PI4K inhibitors, we examined the presence of DP-rings and their susceptibility to KDU691 in ART-resistant gene-edited lines bearing K13 mutations, namely Dd2^R539T^, Dd2^I543T^ and Dd2^C580Y^, as well as in Dd2 K13-WT parasites^11, 12^. Of the three K13 mutations, the C580Y mutation is predominant in western Cambodia; however, the Dd2^C580Y^ recombinant was earlier found to display only moderate survival rates when compared to the other two K13 mutant strains^12^. We first assessed the activity of ART and KDU691 against the three K13 mutants and the parental Dd2-WT strain using the standard 72 hours SYBR-Green dose-response assay^29^ (Supplementary Fig. S4A,B). Dd2-WT and K13 mutant lines proved to be equally susceptible to ART during the 72 hours SYBR Green blood stage assay, as the mutants were only resistant at the ring stages. Likewise, these strains proved to be susceptible to KDU691 at a higher concentration given that this compound was less potent than ART (Supplementary Fig. S4A,B).

**Figure 4:**
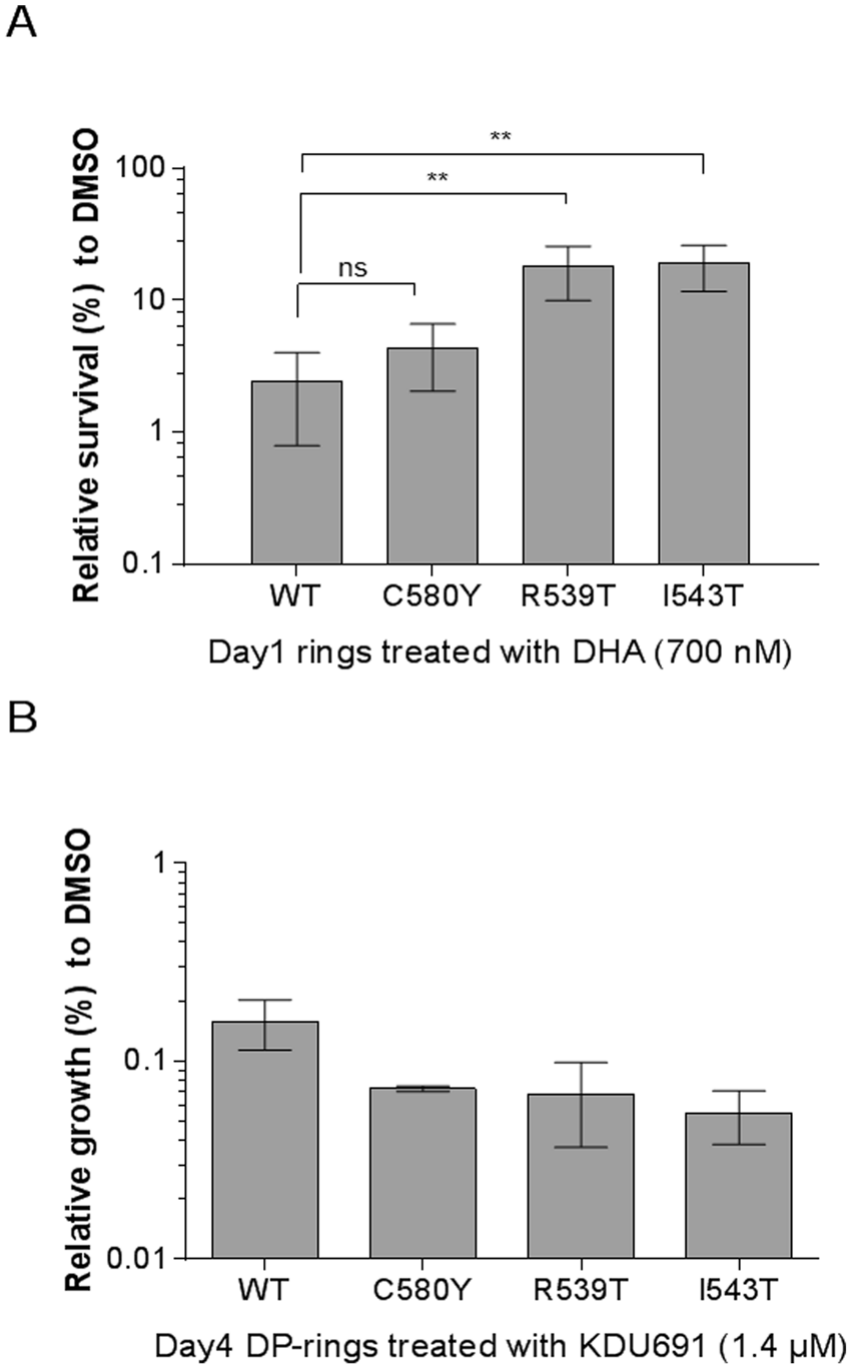
K13-mediated ART resistance is associated with a higher proportion of DP-rings. (**A**) Prevalence of viable DP-rings detected in K13 mutant Dd2 transgenic and Dd2-WT parasites following six hours of exposure to 700 nM DHA. Growth was measured at day one and survival normalized to DMSO-treated control parasites. (**B**) DP-rings susceptibility in K13 mutant Dd2 transgenic and Dd2-WT parasites exposed to KDU691 (1.4 µM) and measured at day four. Data are from three biological experiments and three technical replicates each (means ± SEM % growth). Percent growth was measured by HCI using MitoTracker® Orange relative to DMSO.

**Figure 5:**
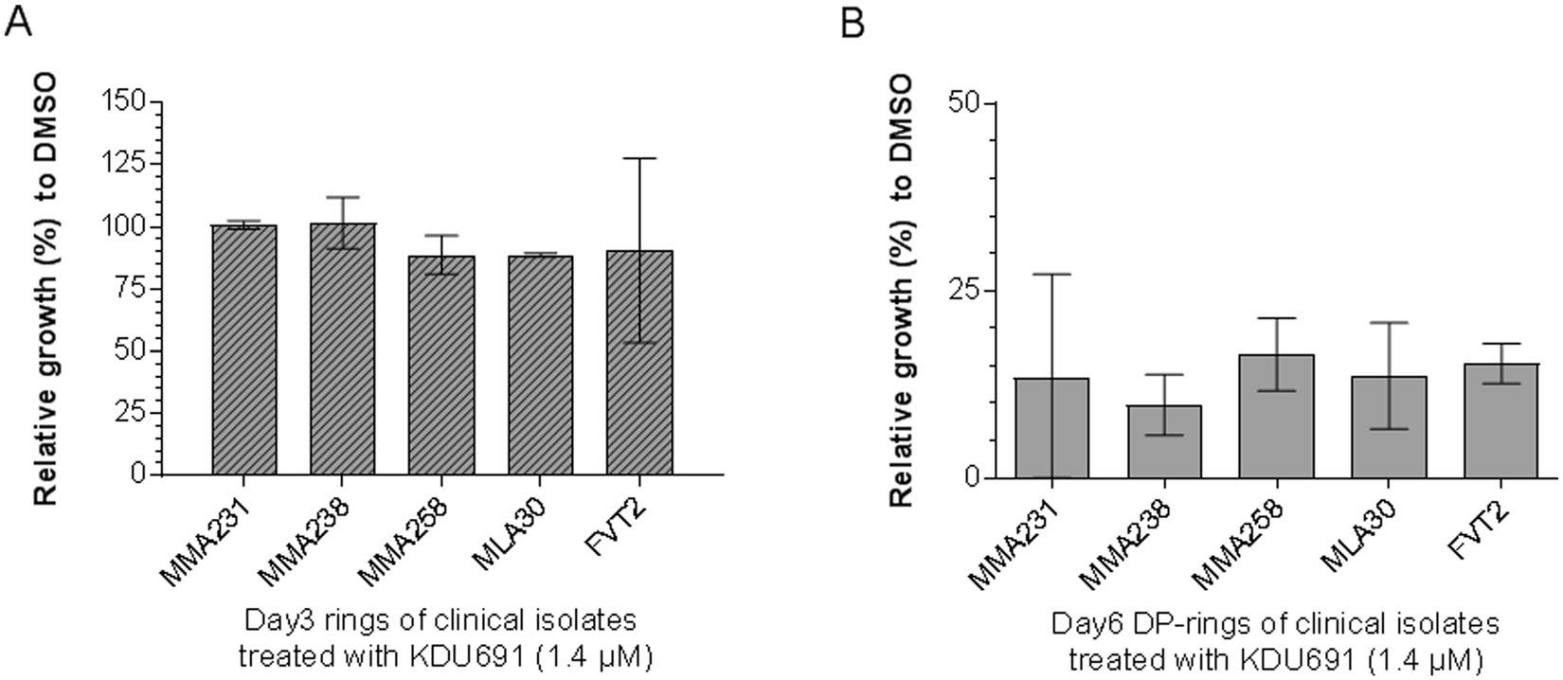
Clinical isolates of DP-rings are susceptible to KDU691. (**A**) Rings and (**B**) DP-rings. Data were normalized to (**A**) DMSO-treated rings and (**B**) DMSO-treated DP-rings. Measurements were taken by HCI using MitoTracker® Orange at day three for rings and day six for DP-rings. KDU691 was added at a concentration of 1.4 µM. Data are from three biological experiments and two technical replicates each (means ± SEM % growth). Percent growth was measured by HCI using MitoTracker® Orange relative to DMSO.

We then assessed the susceptibility to KDU691 (1.4 μM) of 3–6 hours old sorbitol-synchronized rings expressing either mutant K13 or a K13-WT allele. We exposed parasites to a 24 hours pulse of 1.4 µM KDU691 and measured survival/parasitemia on day three (Supplementary Fig. S4C). KDU691 had no inhibitory effect on the rings following 24 hours of exposure in all four Dd2 recombinant strains (Supplementary Fig. S4C). Interestingly, a two- to 10-fold increase in the number of DP-rings in the K13 mutants was observed compared with Dd2-WT (Fig. 4A). However, these DP-rings of K13 mutants proved to be equally susceptible to KDU691 when treated for 24 hours (Fig. 4B). As predicted, TQ remained effective against both rings and DP-rings of Dd2-K13 mutants and parental WT parasites (Supplementary Fig. S4D,E). In experiments using clinical isolates, we obtained similar results demonstrating that KDU691 remain selectively active against DP-rings (Fig. 5 and Supplementary Fig. S5). Together these data suggest that although K13-mediated ART resistance seems to increase the fraction of DP-rings, K13 mutations do not interfere with the ability of KDU691 to selectively kill DP-rings.

## Discussion

Recent molecular and population genetic studies have identified mutations in the K13 gene as a marker of ART-resistance^10, 30, 31^. Prior studies have provided evidence that exposure to ART can induce dormancy, a parasite mechanism that is thought to help survive in the face of drug pressure^16^. In the present study, we used the imidazopyrazine KDU691, a PI4K inhibitor, and the antimalarial drugs TQ, ATQ, and LUM to evaluate the drug susceptibility profiles of DP-rings. KDU691 was previously shown to be active against several stages of the *Plasmodium* life cycle, but was unable to inhibit rings^26^. Here we report that KDU691 selectively kills a small subset of DP-rings but not developing rings. In contrast, ART kills the majority of rings except a small population of surviving DP-rings. TQ is equally active on both.

In agreement with previous studies, KDU691 likely inhibits PI4K in the DP-rings, as mutant parasites encoding the Dd2*-PfPI4K-S1320L* and Dd2-*PfRab11A-D139Y* substitution proved resistant to the PI4K inhibitor. Mutations that confer resistance to KDU691^26^ were also found to be protective in the dormant state as compared to the susceptible WT strains. This finding suggests that PI4K is a potential target for drug development to eliminate DP-rings. Results showed that a significantly larger proportion of rings encoding a K13-mutantion survived DHA treatment (DP-ring). Despite the significant increase in survival rate for the K13 mutant DP-rings, they remained fully susceptible to KDU691. The data suggest that ART and PI4K inhibitors can chemically distinguish normally developing rings from DP-rings.

The recent emergence of ART resistance in Southeast Asia has been convincingly attributed to mutations in the K13 propeller gene^32^. In this study we observed that in the recombinant Dd2-K13 mutant strains we see an increase in the percentage of DP-rings following DHA exposure. Drug tolerance observed in DP-rings has been described as parasites that are refractive to antimalarial drugs during a state of low metabolic activity^16^, a phenomenon common to bacteria^33, 34^ that has yet to be characterized in detail in *Plasmodium*. Here we observe that while DP-rings were tolerant to ATQ, LUM and ART they were susceptible to both TQ and the PI4K inhibitor KDU691. While TQ is currently in late stage clinical development as a prophylactic drug candidate for *P. vivax* malaria, like primaquine it can cause hemolysis in glucose-6-phosphate dehydrogenase-deficient patients, thereby restricting its widespread use. PI4K inhibitors such as the drug candidate MMV39004834 might be more suitable alternatives to target DP-rings in novel ART-based drug combinations.

The function of PI4K during dormancy of the DP-rings has yet to be established. Down-regulation of metabolic and cellular pathways in ring stages appears to be associated with higher resilience to ART, and decelerated development in early ring stages has been shown in ART-resistant clinical isolates^11^. ART-resistant parasite isolates from Southeast Asia bearing the K13 mutation were reported to display an extended ring stage and longer cell cycle^18^. Mutated K13 proteins may interfere with de-ubiquitinylation of transcription factors involved in the regulation of oxidative stress and cell cycle genes, resulting in an extended cell cycle^11, 23, 35–38^. Another lipid kinase, the *P. falciparum* phosphatidylinositol-3-kinase (*Pf*PI3K) has also been proposed to be a mediator of ART resistance whereby the *Pf*PI3K expression is increased through a K13-mediated decrease of ubiquintinylation^35^. Similarly, PI4K might exert a regulatory function in the control or reactivation of the dormant DP-rings and ART resistance through the regulation of phospholipid metabolism.

Experimentally, we have shown that a sub-population of *P. falciparum* rings with a pyknotic appearance can survive or be induced by DHA treatment (DP-rings) and resume growth after the removal of this drug pressure. This study further supports the notion that within the *Plasmodium* ring population, there are at least two forms of parasites, comprising primarily of developing rings susceptible to ART derivatives and a small sub-population of phenotypically DHA-resistant DP-rings. These two ring types can be chemically discriminated by both ART and the PI4K inhibitor. This suggests that metabolic pathways that are not operating in rings control the viability of DP-rings. In conclusion, we provide evidence that *Plasmodium* PI4K inhibitors selectively kill DP-ring stage parasites which suggest that, if combined with ACTs, PI4K inhibitors might be of therapeutic benefit in countries with emerging ART drug resistance.

## Material and Methods

### Antimalarial drugs

All compounds used in the study were obtained from Sigma-Aldrich (St. Louis, MO, USA), except for KDU691 that was synthesized at Novartis^26, 39^.

#### Parasites

This study employed the *P. falciparum* laboratory chloroquine- and pyrimethamine-resistant W2 strain (Indochina), *P. falciparum* Dd2 parental strain (a clone of W2MEF), Dd2*-PI4K-SI320L*, Dd2*-Rab11A-D139Y* transgenic parasite lines resistant to KDU691^26^; and ART-resistant transgenic lines harboring mutations in the *K13* gene^11, 12^, Dd^2R539T^, Dd2^I543T^ and Dd2^C580Y^ and *P. falciparum* clinical isolates (MMA231, MMA238, MMA258, MLA30, and FVT2). The five isolates used in this study were collected under the approved ethical guidelines of the Oxford Tropical Research Ethics Committee (OXTREC 562-15) and the Faculty of Tropical Medicine, Mahidol University (MUTM 2015-019-01).

#### Parasites culture

*P. falciparum* parasites were cultured using standard HEPES-buffered RPMI 1640 medium (Gibco Life Technologies, Singapore) supplemented with 0.5% Albumax and 4% of red blood cells (RBCs) (ORh^+^). RBCs used in culture media were obtained from Innovative Research, USA or the Australian Red Cross Blood Service. At each cycle and prior to drug exposure, parasites were synchronized with 5% D-sorbitol^40^.

#### Susceptibility testing of developing rings and DP-rings

The IC_90_ values of the drugs were determined using a standard SYBR-Green cell proliferation assay with 72 hours durations as previously described^29^. For rings and DP-rings, drug susceptibility testing of three to six hour old rings were used from sorbitol-synchronized cultures. Rhodamine 123 or MitoTracker® Orange viability markers were used for daily assessment of parasites. Dose-response curves for W2 WT rings and DP-rings were generated with sorbitol-synchronized parasites. Briefly, parasites were synchronized once with sorbitol at the ring stage. One sample of 3–6 hours old synchronized rings was directly exposed to the drugs for 22 hours. The second sample of the 3–6 hours synchronized rings was pretreated with DHA (700 nM) for six hours (DP-rings), washed three times to remove compounds and finally exposed to the drugs 18 hours later for 22 hours. Plates were stained with Rhodamine 123 and analyzed by flow cytometry.

#### Susceptibility testing of developing rings

To test the drug susceptibility of rings, synchronized ring-stage cultures at 0.1% parasitemia were treated with TQ, ATQ, LUM and KDU691 at concentrations equivalent to their respective IC_90_ values for either six or 24 hours (Fig. 2A). For the *P. falciparum* W2 strain these concentrations were 700 nM of TQ, 3 nM of ATQ, 60 nM of LUM, 700 nM of KDU691. Concentrations twice the IC_90_ of the drugs were used against Dd2-WT, the K13 mutant transgenic lines, and the *P. falciparum* clinical isolates (MMA231, MMA238, MMA258, MLA30, and FVT2).

#### Susceptibility testing of DP-rings

DP-rings were induced as previously described^13^ by exposing 1% of synchronized ring-stage cultures to 700 nM of DHA for six hours, followed by three washes with 1x Phosphate-buffer saline (PBS) to remove drug and debris. After the last wash, RBC pellets were re-suspended in the original volume of growth media and incubated under normal growth conditions for a further 18 hours. Following this incubation, DP-ring cultures were exposed to TQ, ATQ, LUM or KDU691 at concentrations equivalent to their respective IC_90_ and incubated for a further 24 hours (Fig. 2B). Samples were then subjected to three consecutive washes in 1x PBS and parasite growth was monitored daily. It is possible that low concentrations of compounds such as LUM might persist intracellularly, even after three washes, and affect other stages. After drug exposure the rings and DP-rings were monitored for five and seven days respectively by staining for viability with Rhodamine 123, as previously described^24^ and MitoTracker® Orange (250 nM final concentration in culture media for 24 hours at 37 °C in 5% CO_2_). Either Rhodamine 123^24^ or MitoTracker® Orange dyes were added to the cultures adjusted to 2% hematocrit in Greiner PS Black Cell Culture 96-Well, F-Bottom µClear Plate format (product code: 655090) for high-content imaging. The uptake of Rhodamine 123 and MitoTracker® Orange is dependent on the negative mitochondrial membrane potential and is indicative of cell viability^41^. For the images in Fig. 1A, parasite nuclei were stained with 1 µg/ml of diamidino-phenylindole (DAPI; Sigma) and the mitochondrial stain MitoTracker® Orange (2 µM final), all in culture media for two hours at 37 °C in 5% CO_2_. After two hours staining, the media was removed and 1/500 diluted wheat germ agglutinin (WGA) conjugated with Alexa Fluor® 647 (1 mg/ml stock concentration) was used to stain the red blood cells in 1x PBS for 10 minutes. Cells were then washed twice in 1x PBS. Images were captured using the Opera® confocal high-content screening system (PerkinElmer) at 60x magnification.

#### High-content imaging

(HCI) was carried out on an Opera® system, and the fluorescent dye MitoTracker® Orange was used to monitor parasite growth. The HCI readout used here measured only the absolute number of live parasite counts per field bases on MitoTracker® Orange staining only and not as a percentage of parasitemia.

#### Data analysis

Parasitemias were assessed by HCI on OPERA or by microscopy using Giemsa-stained slides. Dose-response curves and inhibitory concentrations 50% (IC_50_) were calculated by non-linear regression analysis using GraphPad Prism 6 software (with the data normalized to the untreated controls). Kinetic graphs were generated using GraphPad Prism 6. ANOVA Test was used to evaluate P-values. Data are from three independent experiments done in duplicate or triplicate (the latter for the K13 mutant transgenic lines).

## Electronic supplementary material

Supplementary Figures
