# Supplementary material for: The Plasmodium PI(4)K inhibitor KDU691 selectively inhibits dihydroartemisinin-pretreated Plasmodium falciparum ring-stage parasites
Source: Sci Rep. 2017 May 24;7:2325. doi: 10.1038/s41598-017-02440-6 (PMC5443816; doi:10.1038/s41598-017-02440-6)
Supplement: Supplementary file 1 — Supplementary Figures [file 41598_2017_2440_MOESM1_ESM.pdf]

The *Plasmodium* PI(4)K inhibitor KDU691 selectively inhibits dihydroartemisinin-pretreated *Plasmodium falciparum* ring-stage parasites.

L. Dembele[1], X. Ang[1], M. Chavchich[2], G. M.C. Bonamy[1], J. Selva1, M. Yi-Xiu Lim[1], C. Bodenreider[1], B. K. S. Yeung[1], F. Nosten[3,4], B. Russell[5], M. D. Edstein[2], J. Straimer[6], D. A. Fidock[6,7], T. T. Diagana[1], P. Bifani[1,5§]

[1]Novartis Institute for Tropical Diseases, 10 Biopolis Road, #05-01 Chromos, Singapore, 138670

[2]Department of Drug Evaluation, Australian Army Malaria Institute, Brisbane, QLD, 4051, Australia

3Shoklo Malaria Research Unit, Mahidol-Oxford Tropical Medicine Research Unit, Faculty of Tropical Medicine, Mahidol University, Mae Sot, Thailand.

[4]Centre for Tropical Medicine and Global Health, Nuffield Department of Medicine, University of Oxford, Oxford, UK.

[5]Department of Microbiology and Immunology Program, Yong Loo Lin School of Medicine, Life Sciences Institute, National University of Singapore, 119077 Singapore

[6]Department of Microbiology and Immunology, Columbia University Medical Center, New York, NY 10032, USA

[7]Division of Infectious Diseases, Department of Medicine, Columbia University Medical Center, New York, NY 10032, USA

§Correspondence: pablo.bifani@novartis.com

Supplementary Figure 1

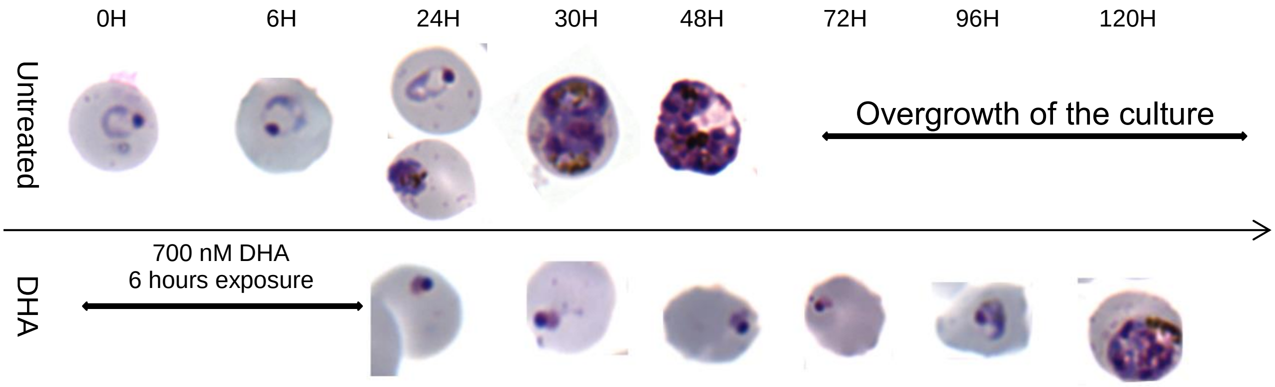

Supplementary Figure 1. *P. falciparum* rings and DP-rings display different morphology. Morphology of the rings and DP-rings in Giemsa-stained slides.

Supplementary Figure 2

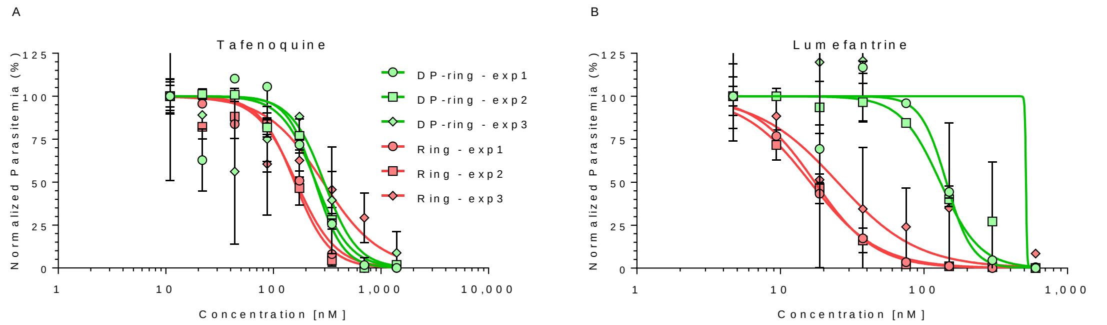

	DP-ring - exp1	DP-ring - exp2	DP-ring - exp3	Ring - exp1	Ring - exp2	Ring - exp3
IC50	245	248	296	163	158	290

	DP-ring - exp1	DP-ring - exp2	DP-ring - exp3	Ring - exp1	Ring - exp2	Ring - exp3
IC50	144	130	~ 514	17	16	25

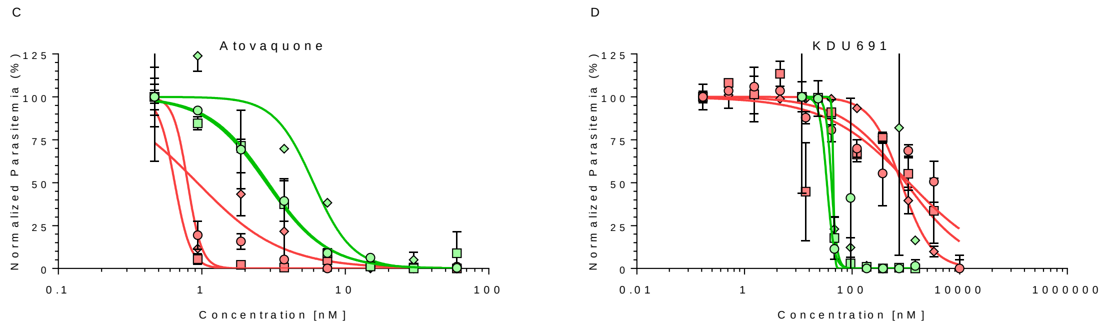

	DP-ring - exp1	DP-ring - exp2	DP-ring - exp3	Ring - exp1	Ring - exp2	Ring - exp3
IC50	2.9	2.8	6	0.8	0.65	0.93

	DP-ring - exp1	DP-ring - exp2	DP-ring - exp3	Ring - exp1	Ring - exp2	Ring - exp3
IC50	34	42	~ 45	1146	994	821

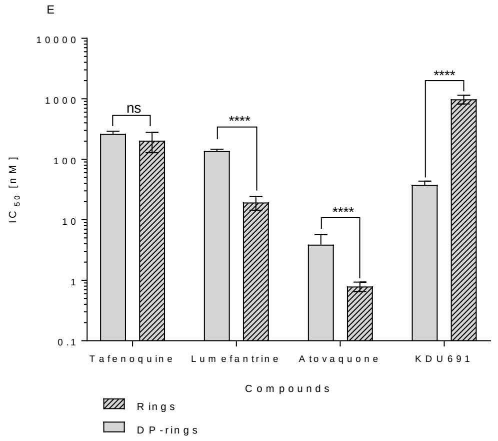

Supplementary Figure 2. Susceptibility testing against W2 WT rings and DP-rings measured by flow cytometry and Rhodamine 123 (IC50 values, nM). Dose response curves of TQ, LUM, ATQ and KDU691 against (A) DP-rings and rings of W2 WT strain measured 22 hours post treatment. Synchronized rings were directly treated with antimalarial for 22 hours for rings. Synchronized rings were the pre-treated with DHA for 6 hours (PD-rings), washed and 18 hours later treated for an additional 22 hours. (B) Comparative antimalarial IC50 values (nM) against rings and DP-rings of the W2 strain. Data were obtained from three independent biological experiments with technical duplicates (means ± SEM). Percent growth was measured by HCI using MitoTracker® Orange relative to DMSO.

Supplementary Figure 3

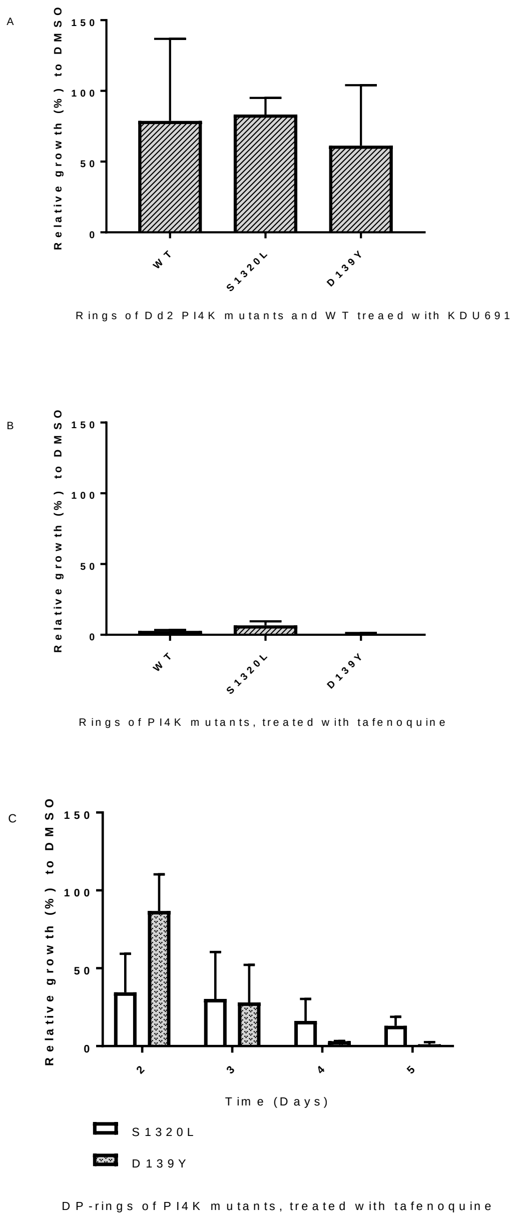

Supplementary Figure 3. Susceptibility testing of PI4K-resistant *P. falciparum* Dd2-*PfPI4K-S1320L* and the Dd2-*PfRab11A-D139Y* strains to KDU691 and tafenoquine. (A) KDU691 (1.4 µM) activity on rings from PI4K-resistant and WT strains measured by HCI and MitoTracker® Orange at day three. (B) TQ (1.4 µM) activity on rings from PI4K resistant strains measured at day three. (C) TQ (1.4 µM) activity on DP-rings from PI4K resistant strains measured at day four. Data are means ± SEM and were obtained from three independent experiments done in triplicate. Percent growth was measured by HCI using MitoTracker® Orange relative to DMSO.

Supplementary Figure 4

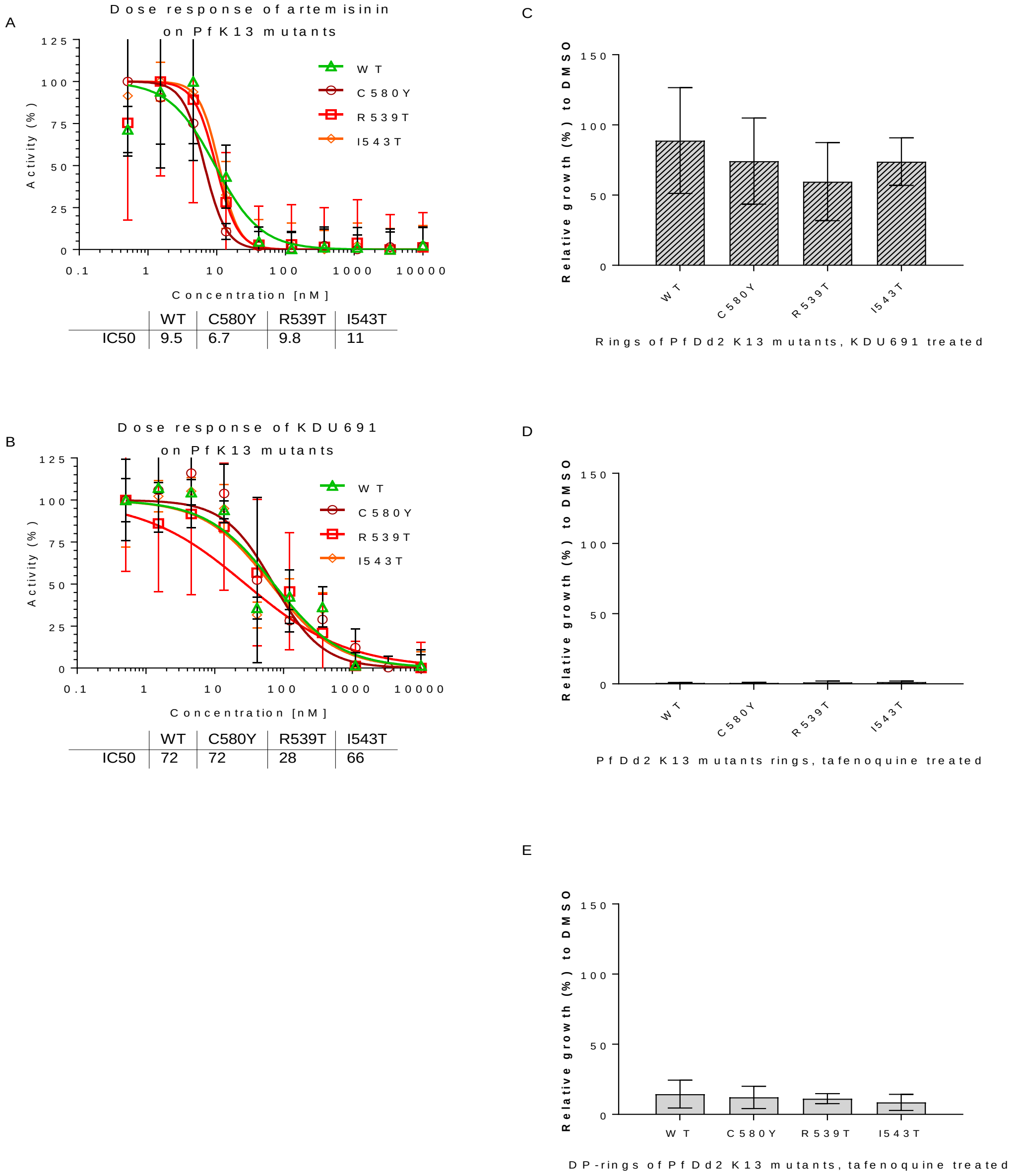

	WT	C580Y	R539T	I543T
IC50	9.5	6.7	9.8	11

	WT	C580Y	R539T	I543T
IC50	72	72	28	66

Supplementary Figure 4. Susceptibilities of laboratory adapted Dd2 *Pf*Kelch 13 mutants and wild-type (WT) strains to artemisinin, KDU691 and TQ measured by HCI and MitoTracker® Orange. IC50 values of (A) Artemisinin and (B) KDU691 against the Dd2-K13 mutants and WT strains. Values were determined by the SYBR Green cell proliferation assay at 72 hours. (C) Susceptibility of rings of the Dd2 K13 mutant transgenic strains and Dd2-WT strain to KDU691 (1.4 µM) at day three. (D) TQ (1.4 µM) activity against rings of Dd2-K13 mutant transgenic lines and Dd2-WT strains at day three. (E) TQ (1.4 µM) activity against DP-rings of Dd2-K13 mutant transgenic lines and Dd2-WT at day four. Data were obtained from three independent biological experiments with technical duplicates (means ± SEM). Percent growth was measured by HCI using MitoTracker® Orange relative to DMSO.

Supplementary Figure 5

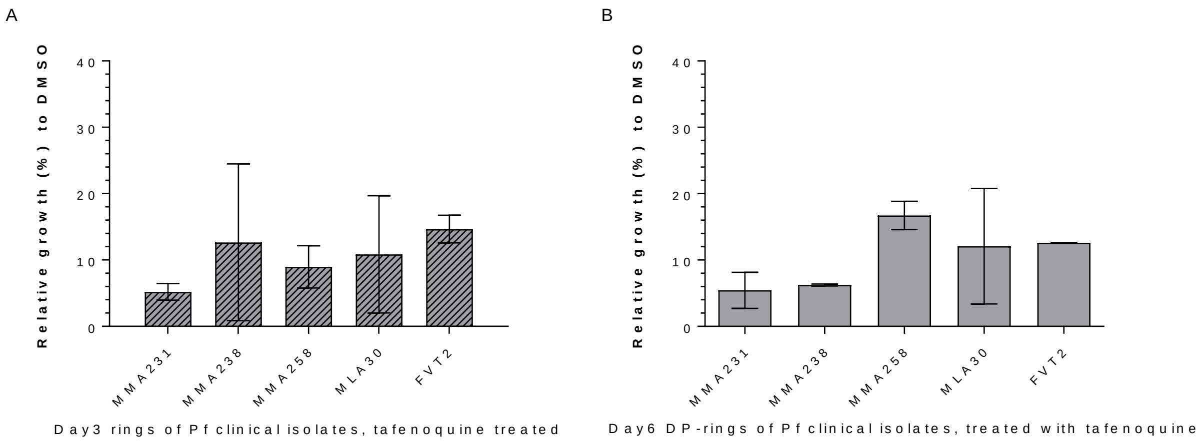

Supplementary Figure 5. Susceptibility of clinical *P. falciparum* isolates to TQ measured by HCI and MitoTracker® Orange. (A) Rings and (B) DP-rings of *P. falciparum* clinical isolates susceptibility to TQ measured respectively at day three and day six. Data were obtained from three independent biological experiments with technical duplicates (means ± SEM). Percent growth was measured by HCI using MitoTracker® Orange relative to DMSO.
